# Allosteric mechanism of action of the therapeutic anti-IgE antibody omalizumab

**DOI:** 10.1074/jbc.M117.776476

**Published:** 2017-04-24

**Authors:** Anna M. Davies, Elizabeth G. Allan, Anthony H. Keeble, Jean Delgado, Benjamin P. Cossins, Alkistis N. Mitropoulou, Marie O. Y. Pang, Tom Ceska, Andrew J. Beavil, Graham Craggs, Marta Westwood, Alistair J. Henry, James M. McDonnell, Brian J. Sutton

**Affiliations:** From the ‡ Randall Division of Cell and Molecular Biophysics, King's College London, New Hunt's House, Guy's Campus, London SE1 1UL,; the § Medical Research Council and Asthma UK Centre in Allergic Mechanisms of Asthma, London SE1 1UL, and; ¶ UCB-Celltech, 208 Bath Road, Slough SL1 3WE, United Kingdom

**Keywords:** allergy, allosteric regulation, antibody, immunoglobulin E (IgE), X-ray crystallography, omalizumab

## Abstract

Immunoglobulin E and its interactions with receptors FcϵRI and CD23 play a central role in allergic disease. Omalizumab, a clinically approved therapeutic antibody, inhibits the interaction between IgE and FcϵRI, preventing mast cell and basophil activation, and blocks IgE binding to CD23 on B cells and antigen-presenting cells. We solved the crystal structure of the complex between an omalizumab-derived Fab and IgE-Fc, with one Fab bound to each Cϵ3 domain. Free IgE-Fc adopts an acutely bent structure, but in the complex it is only partially bent, with large-scale conformational changes in the Cϵ3 domains that inhibit the interaction with FcϵRI. CD23 binding is inhibited sterically due to overlapping binding sites on each Cϵ3 domain. Studies of omalizumab Fab binding in solution demonstrate the allosteric basis for FcϵRI inhibition and, together with the structure, reveal how omalizumab may accelerate dissociation of receptor-bound IgE from FcϵRI, exploiting the intrinsic flexibility and allosteric potential of IgE.

## Introduction

Immunoglobulin E (IgE) antibodies play a crucial role in allergic disease, binding to allergens through their Fab arms and expressing their effector functions by binding to receptors for the Fc region (1). The two principal IgE receptors are FcϵRI and CD23/FcϵRII, commonly referred to as the high- and low-affinity receptors, respectively. On mast cells and basophils, IgE binds to FcϵRI so tightly (*K_D_* ≈10^−10^ m) that such cells are sensitized with pre-bound IgE, requiring only the presence of an allergen to cross-link IgE/FcϵRI complexes and elicit an immediate reaction. CD23 is a homotrimer, and thus the intrinsically lower affinity of each IgE-binding C-type lectin-like “head” domain (*K_D_* ≈10^−7^ m) can be enhanced by an avidity effect when binding to aggregated IgE in immune complexes, nearly matching that of FcϵRI for IgE (2). CD23 expressed on B cells is involved in IgE regulation, and expression on airway and gut epithelial cells mediates transcytosis of IgE/allergen complexes (1, 2). FcϵRI and CD23 are also both expressed on a range of antigen-presenting cells. Thus IgE-receptor interactions are involved in multiple aspects of the allergic response, and IgE is a long-standing target for therapeutic intervention (3).

The Fc region of IgE comprises a disulfide-linked dimer of three domains: Cϵ2, Cϵ3, and Cϵ4. Early FRET studies of a chimeric IgE (4, 5), and X-ray solution scattering studies of IgE-Fc (6), indicated a compact, bent structure, and the crystal structure of IgE-Fc later revealed an acutely and asymmetrically bent conformation, with the (Cϵ2)_2_ domain pair folded back onto the Cϵ3 and Cϵ4 domains (7). The bend, defined as the angle between the local 2-fold axis of the (Cϵ2)_2_ domain pair and that of Fcϵ3–4 (the region comprising only the Cϵ3 and Cϵ4 domains), was found to become even more acute in the crystal structure of IgE-Fc bound to sFcϵRIα, the soluble extracellular domains of the IgE-binding α-chain of the receptor (8). FRET studies with N- and C-terminally labeled IgE-Fc confirmed this enhanced bend upon sFcϵRIα binding (9).

The FcϵRI-binding site spans both Cϵ3 domains in the Cϵ2-proximal region (8, 10), although the Cϵ2 domain is not directly involved; the engagement of both chains accounts for the 1:1 binding stoichiometry. In contrast, two CD23 molecules bind to IgE-Fc, one in each chain, and at the other Cϵ4-proximal end of the Cϵ3 domain (11–14). CD23 binding also causes a conformational change in IgE-Fc (14), but not one that significantly affects the bend (9). However, the relatively “closed” disposition of the Cϵ3 domains in the complex with the soluble head domain of CD23 (sCD23), compared with free IgE-Fc, is incompatible with the more “open” arrangement of these domains that is required for FcϵRI binding. This partly explains the mutual exclusion of FcϵRI and CD23 binding (11, 12), although other factors such as local conformational changes and modifications of conformational dynamics (15) also likely contribute to the allosteric communication between the two receptor-binding sites (2).

A more extreme degree of flexibility in IgE-Fc was recently discovered through studies of a complex with an anti-IgE-Fc Fab, termed aϵFab (16). Two aϵFab molecules bind to IgE-Fc in a symmetrical manner, one on each Cϵ3 domain, trapping a fully extended conformation in which the local 2-fold axes of the (Cϵ2)_2_ domains and Fcϵ3–4 region are virtually coincident. Analysis of the complex formation in solution, together with molecular dynamics simulations of free IgE-Fc, suggests that the (Cϵ2)_2_ domain pair could “flip” over from one side of the Fcϵ3–4 region to the other (16). The IgE-Fc conformation stabilized by this anti-IgE antibody is incompatible with FcϵRI binding, explaining its inhibitory activity (16).

Omalizumab is an anti-IgE monoclonal IgG1 antibody that is approved for therapeutic use (Xolair®, Novartis) (17). It binds to free IgE and inhibits both FcϵRI and CD23 binding. The site of binding had been mapped to the Cϵ3 domain by peptide inhibition and molecular modeling and was recently confirmed by a crystal structure (18–20). Recently, an inhibitor was discovered that actively disrupted preformed IgE/FcϵRI complexes: a Designed Ankyrin Repeat Protein (DARPin) was found to bind to the Cϵ3 domain of receptor-bound IgE and accelerate its dissociation from FcϵRI (21). The crystal structure of the 2:1 complex of this DARPin (DARPin E2_79) with an Fcϵ3–4 molecule constrained by an engineered disulfide bond (G335C), which artificially locks the Cϵ3 domains into a closed conformation, revealed the nature and location of the binding site but left its mechanism of action unclear. It was subsequently reported that omalizumab could also facilitate dissociation of FcϵRI-bound IgE, although only at very high concentrations that were substantially greater than those utilized therapeutically (22, 23). Omalizumab binding to FRET-labeled IgE-Fc indicated a slight degree of unbending (9) and the potential for allosteric rather than direct inhibition of FcϵRI binding. The recent crystal structure of an omalizumab Fab complex is with the same Fcϵ3–4 molecule present in the DARPin complex (20); this constrained Fcϵ3–4 construct lacks the Cϵ2 domains and thus cannot report on unbending or other conformational changes.

We report here the crystal structure of the complex between IgE-Fc and a Fab derived from omalizumab. The structure of the complex reveals substantial conformational changes in IgE-Fc, revealing the mechanism of action of omalizumab, both for receptor inhibition and accelerated dissociation of IgE from FcϵRI. Solution studies demonstrate that these mechanisms exploit the intrinsic flexibility of IgE.

## Results

Despite extensive efforts, crystallization trials for IgE-Fc in complex with the omalizumab Fab resulted in selective crystallization of the Fab fragment only. Others have reported a similar failure to crystallize the complex with IgE-Fc (24). The recently reported structure for the omalizumab Fab complex (20) is with an Fcϵ3–4 molecule that contains a G335C mutation; this mutation artificially locks the Cϵ3 domains into a closed conformation. We designed a Fab, derived from omalizumab, with three point mutations, two in the V_L_ domain framework region (S81R and Q83R) and one in the Cκ domain (L158P) (supplemental Fig. S1), with the purpose of disrupting favorable crystal contacts observed in the omalizumab Fab crystal structure. We term this omalizumab-derived Fab FabXol3.

### Overall structure of the FabXol3/IgE-Fc complex

**Figure 1.**
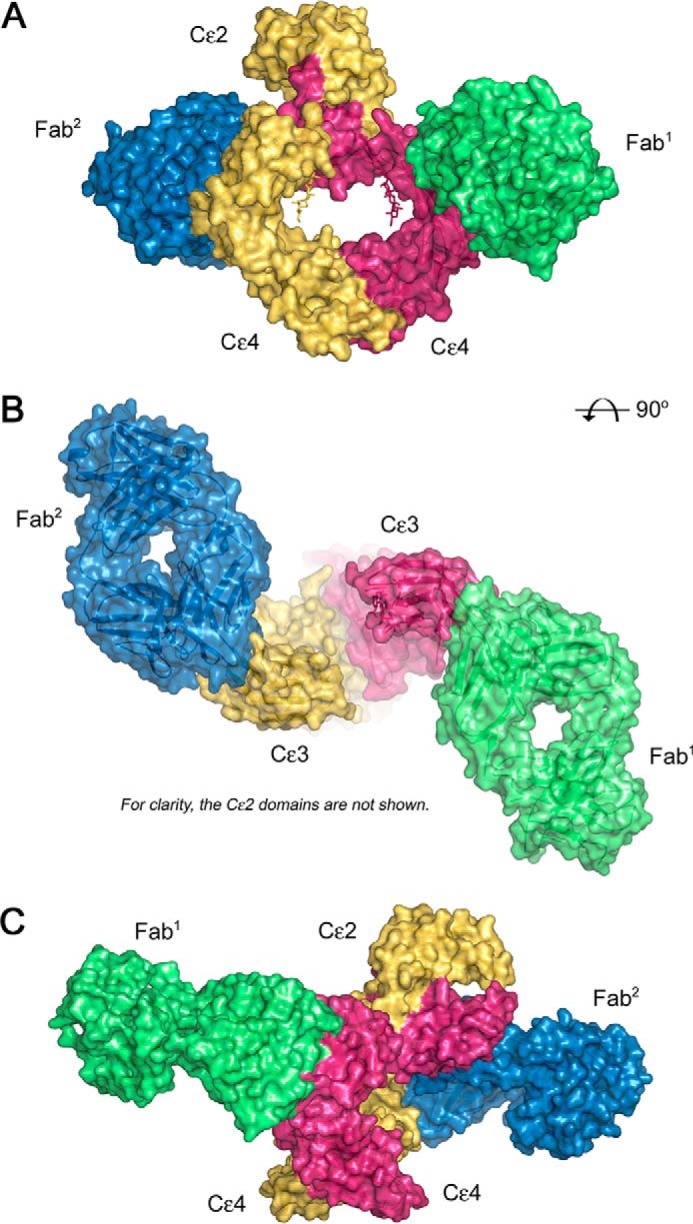
**Overall structure of IgE-Fc in complex with FabXol3.** *A*, FabXol3 binds to IgE-Fc with 2:1 stoichiometry. Fab^1^ (*green*) engages IgE-Fc chain B (*pink*) exclusively through the Cϵ3 domain. Fab^2^ (*blue*) interacts with IgE-Fc chain A (*yellow*) through the Cϵ3 domain and forms a minor interaction with the Cϵ2 domain from IgE-Fc chain B (*pink*). *B*, two Fabs form a pseudo-symmetric complex about the 2-fold axis of the Fcϵ3–4 region. For clarity, the Cϵ2 domains are not shown. *C*, IgE-Fc is asymmetrically bent in the FabXol3 complex. The Cϵ2 domain from chain B (*pink*) contacts Fab^2^ (*blue*).

**Figure 2.**
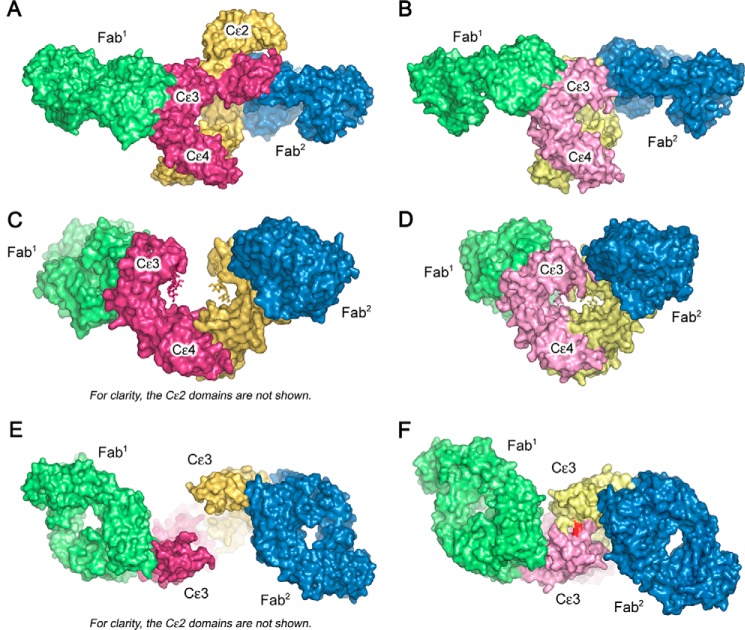
**Comparison of the FabXol3/IgE-Fc and omalizumab Fab/Fcϵ3–4 complexes.** *A*, side view of IgE-Fc (*yellow* and *pink*) in the FabXol3 (*green* and *blue*) complex, showing the asymmetric bend in IgE-Fc. *B*, side view of the constrained Fcϵ3–4 molecule (*pale yellow* and *pink*) in the omalizumab Fab (*green* and *blue*) complex (20). *C*, front view of IgE-Fc in the FabXol3 complex (90° clockwise rotation from the view shown in *A*). For clarity, the (Cϵ2)_2_ domain pair is not shown. *D*, front view of the constrained Fcϵ3–4 molecule in the omalizumab Fab complex (90° clockwise rotation from the view shown in *B*). *E*, top view of IgE-Fc in the FabXol3 complex (90° rotation toward the reader from the view shown in *C*). For clarity, the (Cϵ2)_2_ domain pair is not shown. *F*, top view of the constrained Fcϵ3–4 molecule in the omalizumab Fab complex (90° rotation toward the reader from the view shown in *D*). The position of the engineered disulfide bond that locks the Cϵ3 domains into a closed conformation is colored *red*.

We determined the crystal structure of the complex between IgE-Fc and FabXol3 to 3.7 Å resolution (Fig. 1 and supplemental Movie S1). Two FabXol3 molecules (Fab^1^ and Fab^2^) bind to an asymmetric, partially bent IgE-Fc molecule, and the Fab engages one edge of the exposed face of each Cϵ3 domain (Fig. 1). Fab^1^ engages the Cϵ3 domain of IgE-Fc chain B, whereas Fab^2^ engages the Cϵ3 domain of IgE-Fc chain A. Because of the partially bent conformation of IgE-Fc in the complex, the light chain of Fab^2^ also forms a minor interaction with the Cϵ2 domain from IgE-Fc chain B (see supplemental data for details of this interaction). The overall structure of IgE-Fc in complex with FabXol3 is compared with that of the constrained Fcϵ3–4 molecule in complex with the omalizumab Fab (20) in Fig. 2. The FabXol3/IgE-Fc complex not only reveals the effect of omalizumab binding on the position of the (Cϵ2)_2_ domain pair (Fig. 2*A*), which is absent in the Fcϵ3–4 molecule (Fig. 2*B*), but also shows that the Cϵ3 domains adopt a markedly open conformation (Fig. 2, *C* and *E*), one that cannot be adopted by the disulfide-bonded Cϵ3 domains in the Fcϵ3–4 complex (Fig. 2, *D* and *F*). The bending of the (Cϵ2)_2_ domain pair and the opening of the Cϵ3 domains are described in detail below.

### Interface between IgE-Fc and FabXol3

**Figure 3.**
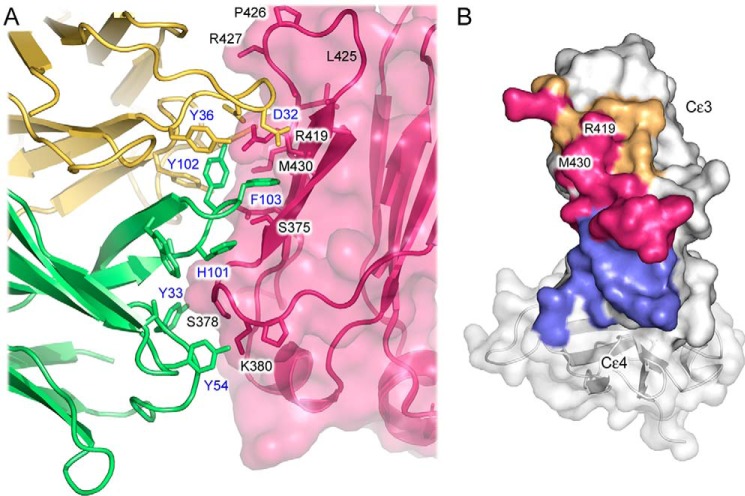
*A*, interface between FabXol3 and IgE-Fc. The interface between FabXol3 Fab^2^ (heavy and light chains colored in *green* and *yellow*, respectively) and the Cϵ3 domain from IgE-Fc (*pink*) is shown. FabXol3 and Cϵ3 domain residue labels are colored *blue* and *black*, respectively. The interface includes hydrogen bonds and van der Waals interactions. A notable feature of the interface is a cation/π interaction between Arg-419 (Cϵ3 domain) and Phe-103 (FabXol3 CDRH3). The Phe-103 side chain is mostly buried in a pocket created by Thr-373, Trp-374, Ser-375, Gln-417, and Arg-419 (Cϵ3 domain). *B*, FabXol3 and DARPin E2_79 (21) bind to an overlapping interface on the Cϵ3 domain. IgE-Fc residues, which only form part of the FabXol3 interface, are colored *orange*, and those that only form part of the DARPin E2_79 interface, which includes part of the Cϵ3-Cϵ4 linker, are colored in *blue*. IgE-Fc residues colored in *pink*, which include Arg-419 and Met-430, are common to both FabXol3 and DARPin E2_79 interfaces.

Each FabXol3 molecule engages one edge of the exposed face of the Cϵ3 domain (C, C′, F, and G strands and base of the FcϵRI receptor-binding FG loop). The interface with IgE-Fc is similar to that reported for the constrained Fcϵ3–4 molecule (20). Both the heavy and light chain of FabXol3 are involved, the former contributing ∼60% to an interface area of ∼715 Å^2^ (Figs. 1 and 3*A* and supplemental Movie S1).

The FabXol3 heavy chain contacts, which differ slightly between the two interfaces, may be summarized as follows: Gly-32 and Tyr-33 (CDRH1) form van der Waals interactions with Ala-377 and Ser-378 (Cϵ3), whereas Tyr-54 (CDRH2) contacts Gly-379–Pro-381 (Cϵ3). The CDRH3 residues contribute the largest contact area and undergo a significant conformational change upon complex formation, when compared with unbound Fab structures (19, 20, 24). CDRH3 residues Ser-100, His-101, Tyr-102, and Trp-106 all form van der Waals interactions with Cϵ3 domain residues that include Ser-375–Gly-379, Gln-417, and Arg-419 (Cϵ3). However, the most striking feature of this part of the interface is the interaction with Phe-103 (CDRH3). Phe-103 is mostly buried in a pocket created by Thr-373, the Trp-374 main chain, Ser-375, Gln-417, and Arg-419 (Cϵ3), and it forms a cation/π-stacking interaction with Arg-419 (Fig. 3*A*).

Arg-419 also plays a key role in the interaction with the FabXol3 light chain (Fig. 3*A*). Arg-419 is within hydrogen-bonding distance of the Tyr-31 (CDRL1) and Asp-32 (CDRL1) main chain carbonyl oxygen atoms, in addition to contacting the Asp-32, Asp-34, and Tyr-36 side chains (forming a hydrogen bond with the Tyr-36 hydroxyl group). Asp-32 also forms van der Waals interactions with Thr-373 and Thr-421 (Cϵ3). By contrast, only two CDRL2 residues contribute to the interface, Tyr-53 (CDRL2) contacts Gln-417 (Cϵ3), and both Tyr-53 and Tyr-57 form van der Waals interactions with Met-430 (Cϵ3); Tyr-57 also forms a hydrogen bond with the Met-430 backbone. As for the heavy chain interaction, there are slight differences in the light chain contacts for Fab^1^ and Fab^2^.

### Comparison of the FabXol3 interface with other anti-IgE complexes

The binding sites on the Cϵ3 domain for FabXol3 and the recently described DARPin E2_79 (21) overlap (Fig. 3*B*) and are of similar size at ∼715 and ∼753 Å^2^, respectively. The Cϵ3 domain residues shared between the two interfaces include Ser-375–Gly-379, Gln-417, Arg-419, Arg-427, and Met-430, but although FabXol3 forms more intimate contacts with the receptor-binding Cϵ3 FG loop, the DARPin E2_79 interface extends in the opposite direction to include the Cϵ3–4 domain linker.

**Figure 4.**
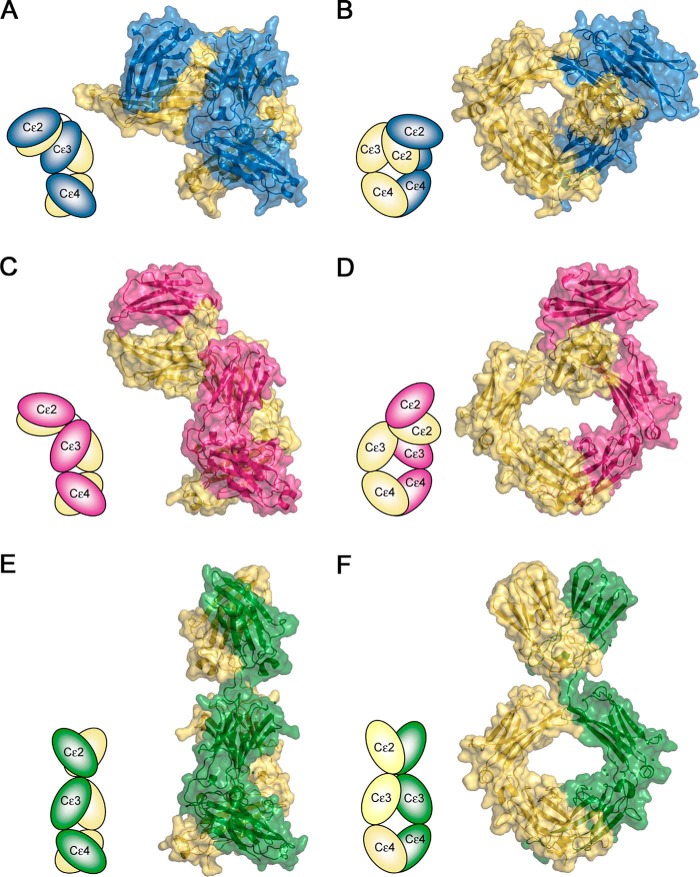
**Conformational flexibility in IgE-Fc.** *A*, side view of free IgE-Fc (8) showing its acute asymmetric bend. *B*, front view of free IgE-Fc (90° anti-clockwise rotation from the view shown in *A*). *C*, side view of IgE-Fc from the FabXol3 complex, revealing a partially bent conformation. *D*, front view of IgE-Fc in the FabXol3 complex (90° anti-clockwise rotation from the view shown in *C*). *E*, side view of fully extended IgE-Fc captured by an anti-IgE-Fc Fab (aϵFab) (16). *F*, front view of extended IgE-Fc (90° anti-clockwise rotation from the view shown in *E*).

The overlapping binding sites of FabXol3 and DARPin E2_79 differ markedly from the interface recently described for the anti-IgE-Fc aϵFab, which captured IgE-Fc in a fully extended conformation (Fig. 4) (16). Not only is the aϵFab interface area approximately double that of FabXol3 and DARPin E2_79, at ∼1400 Å^2^, but aϵFab engages IgE-Fc at a site centered on Arg-393 in Cϵ3, and it also contacts residues in the Cϵ2 domain and the Cϵ2-Cϵ3 linker (16). The crystal structure of another anti-IgE antibody Fab, MEDI4212, in a 2:1 complex with Fcϵ3–4 reveals yet another site for antibody engagement within the Cϵ3 domain, this one involving the *N*-linked oligosaccharide moiety at Asn-394 (25).

### IgE-Fc adopts a partially bent conformation when bound to FabXol3

IgE-Fc is predominantly bent in solution (4–6, 9, 26–28), and the crystal structure for free IgE-Fc revealed an acutely bent (62°) asymmetric conformation, in which the (Cϵ2)_2_ domain pair folded back onto the Cϵ3 and Cϵ4 domains (Fig. 4, *A* and *B*), the Cϵ2 domain of one chain (chain B) contacting the Cϵ4 domain of the other (chain A) (7, 8). IgE-Fc becomes even more acutely bent (54°) upon FcϵRIα engagement (8, 9), and the associated conformational changes involve rotation of the Cϵ3 domain of chain A together with the (Cϵ2)_2_ domain pair, as a rigid unit, away from the Cϵ3 domain of chain B (8).

In contrast to the aϵFab complex, in which IgE-Fc adopts a fully extended, linear conformation (16), IgE-Fc adopts a partially bent conformation in the FabXol3 complex (Figs. 1*C* and 4, *C–F*, and Movies S1 and S2). The site to which Fab^1^ binds is exposed in free, acutely bent IgE-Fc, but further unbending of IgE-Fc, to just over 90°, is required to render the site occupied by Fab^2^ accessible. This unbending of IgE-Fc in the FabXol3 complex is associated with opening of both Cϵ3 domains to create an almost symmetrical Fcϵ3–4 region (Figs. 1*B* and 4, *C* and *D*).

In a recent molecular dynamics simulation exploring unbending of IgE-Fc to an extended structure, it was found that although the acutely bent conformation observed in the crystal structure of free IgE-Fc occupied the lowest energy basin, another distinct and well defined energy basin, corresponding to partially bent IgE-Fc conformations, was observed (16). The partially bent conformation adopted by IgE-Fc in the FabXol3/IgE-Fc complex occupies this particular energy basin (supplemental Fig. S2).

**Figure 5.**
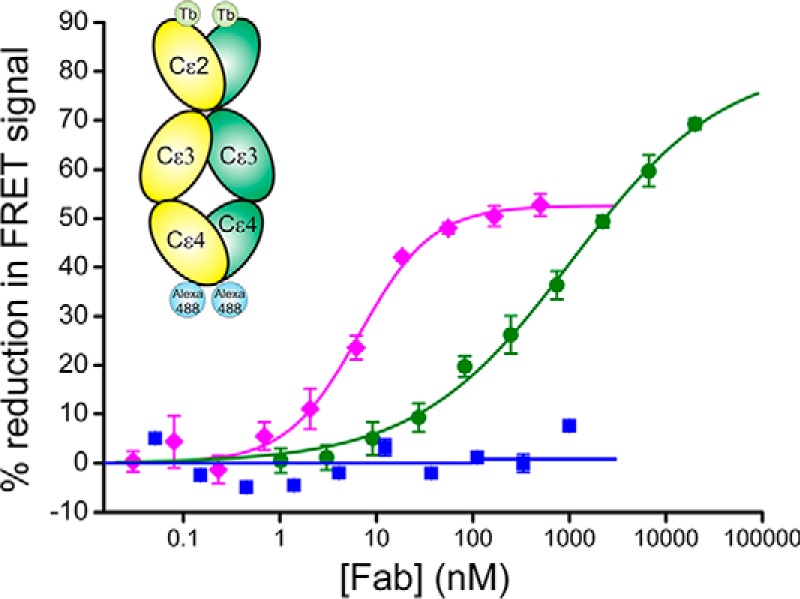
**Effect of anti-IgE Fabs on IgE-Fc conformation measured by FRET.** The FRET ratio (*E*_520_/*E*_485_) was measured in the presence of different concentrations of anti-IgE Fabs, either FabXol3 (*magenta*), aϵFab (*green*), or control Fab (*blue*). aϵFab has previously been shown to fully unbend IgE-Fc (16); the control Fab binds to the Cϵ2 domain of IgE-Fc and does not cause unbending of the molecule.

To test whether we could observe these conformational changes in solution, we performed intramolecular FRET measurements with IgE-Fc labeled with donor and acceptor fluorophores in the Cϵ2 domains and at the C termini, respectively. We have previously shown that binding of aϵFab results in a nearly fully extended IgE-Fc structure (16); data here indicate a >90% reduction of the FRET signal (*E*_max_ = 91%, IC_50_ = ∼1 μm) when two molecules of aϵFab are bound to IgE-Fc (Fig. 5). In contrast, FabXol3, despite its higher affinity, shows only about 50% reduction of the intramolecular FRET signal under saturating conditions (*E*_max_ = 52%, IC_50_ = ∼7 nm). These experiments support the hypothesis that binding of FabXol3 induces large-scale conformational changes in IgE-Fc, consistent with a partially bent conformation.

### Cϵ3 domains adopt a markedly open conformation in the FabXol3/IgE-Fc complex

In crystal structures of IgE-Fc and the Fcϵ3–4 sub-fragment, the Cϵ3 domains adopt a range of different orientations (7, 8, 10, 11, 13, 14, 16, 25, 29), a property associated with allosteric regulation of IgE binding to its two principal receptors, FcϵRI and CD23 (8, 11–14). Both the distance between the Cϵ3 domains and their positions with respect to the Cϵ4 domains have been used to describe the variety of conformations observed for the Fcϵ3–4 region (29) (a full description for these measurements is provided in supplemental data). In the FabXol3/IgE-Fc complex, the Cϵ3 domains are positioned further away from one another than in any other crystal structure containing IgE-Fc or Fcϵ3–4 and thus adopt the most open conformation observed thus far (Figs. 1*B* and 4, *C* and *D*, and supplemental Movie S2); this conformation is significantly more open than the conformation for FcϵRI-bound IgE-Fc (supplemental Fig. S3).

### Effect of FabXol3 on FcϵRI and CD23 receptor binding

**Figure 6.**
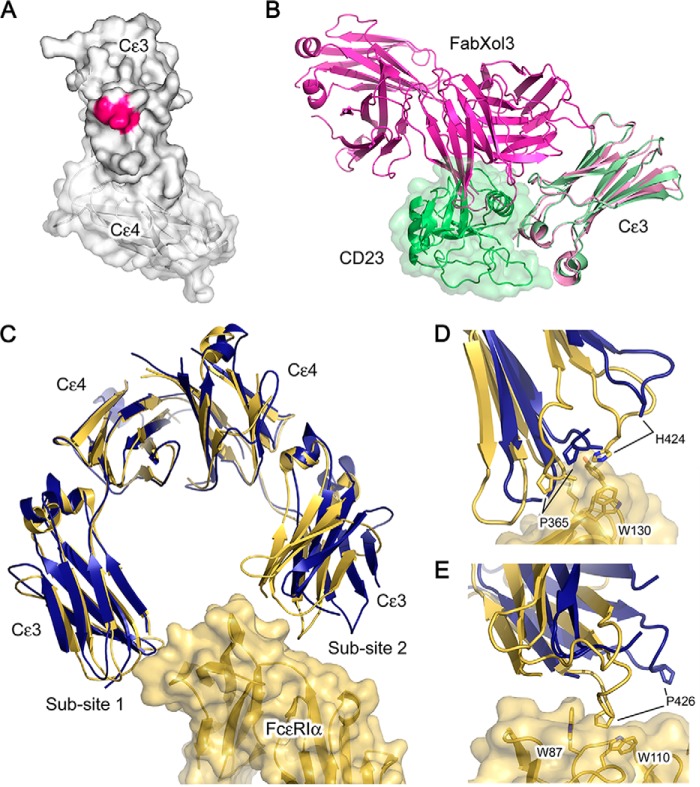
**Disruption of the interaction between IgE-Fc and CD23 and between IgE-Fc and FcϵRI.** *A*, Cϵ3 domain residues that are common to both FabXol3 and CD23 interfaces are colored *pink. B*, superposition of the Cϵ3 domains from the FabXol3/IgE-Fc complex (*light pink*) and the previously reported crystal structure of CD23 in complex with Fcϵ3–4 (11) (*light green*) reveals clashes between CD23 (*green*) and FabXol3 (*dark pink*). The CD23/Fcϵ3–4 complex structure (11) is with an IgE-Fc construct (Fcϵ3–4) that lacks the (Cϵ2)_2_ domain pair. For clarity, the Cϵ4 domains are not shown. *C*, in the FabXol3 complex, the Cϵ3 domains adopt the most open conformation reported thus far for IgE-Fc, which precludes engagement with FcϵRIα. The structure of IgE-Fc in complex with sFcϵRIα (8) is colored *yellow*, and the two sub-sites of receptor engagement are indicated. The structure of FabXol3 in complex with IgE-Fc (*blue*) was superposed on the Cϵ4 domains. *D*, positions of Pro-365 and His-424 at sub-site 1 are indicated to highlight the different positions adopted by the Cϵ3 domains. *E*, substantial displacement of Pro-426 in the FabXol3 complex prevents engagement of the proline sandwich at sub-site 2.

Omalizumab inhibits not only the interaction between IgE-Fc and FcϵRI, but also the interaction between IgE-Fc and CD23 (30). Consistent with the latter, superposition on the Cϵ3 domains from the FabXol3/IgE-Fc structure, and the previously reported structure of CD23 in complex with an Fcϵ3–4 molecule (11), reveals steric clashes between FabXol3 and CD23 at both sites of CD23 engagement on the Cϵ3 domain. Furthermore, Cϵ3 domain residues Arg-376, Ser-378, and Lys-380 are involved in both FabXol3 and CD23 binding (Fig. 6, *A* and *B*) (11, 13, 14, 31). Thus, omalizumab inhibits CD23 binding by orthosteric blocking.

In contrast to CD23 binding to IgE, FcϵRIα binds across both Cϵ3 domains. However, in the FabXol3/IgE-Fc complex, the Cϵ3 domains adopt a conformation that is more open than in FcϵRI-bound IgE-Fc, which precludes simultaneous engagement of both chains (Fig. 6, *C–E*). Moreover, superposition of the FabXol3/IgE-Fc and sFcϵRIα/IgE-Fc (8) complexes reveals potential steric clashes; for example, FabXol3 would clash with the (Cϵ2)_2_ domain pair from the acutely bent conformation found in FcϵRIα-bound IgE-Fc.

Also in contrast to CD23, the binding sites for omalizumab and FcϵRIα do not actually overlap, although FabXol3 CDRL1 residues are positioned immediately adjacent to the FcϵRIα-binding Cϵ3 domain FG loop. This loop, in chain B, contributes to a hydrophobic “proline sandwich” interaction, in which Pro-426 in Cϵ3 packs between two tryptophan residues of FcϵRIα (Fig. 6*E*). Asp-32 (CDRL1) contacts Thr-421; Gly-33 (CDRL1) contacts Pro-426, Arg-427, and Ala-428; and Asp-34 (CDRL1) contacts Arg-427 and Ala-428. These interactions alter the position of the Cϵ3 domain FG loop and would further compromise the binding of IgE to FcϵRI.

Thus, omalizumab binding stabilizes a conformation of IgE-Fc, which is incompatible with FcϵRI binding.

### Interaction of FabXol3 with IgE-Fc in solution

Recently, binding of omalizumab to FcϵRIα-bound Fcϵ3–4 has been reported (22), although it is difficult to see how omalizumab might be able to engage FcϵRI-bound IgE based on the static crystal structures of IgE-Fc in complex with sFcϵRIα (8) and FabXol3. We therefore studied the solution state binding of FabXol3 to IgE-Fc, and we characterized the interaction between FabXol3 and the IgE-Fc/FcϵRI complex. Our results provide insights into the mechanism of action of omalizumab.

**Figure 7.**
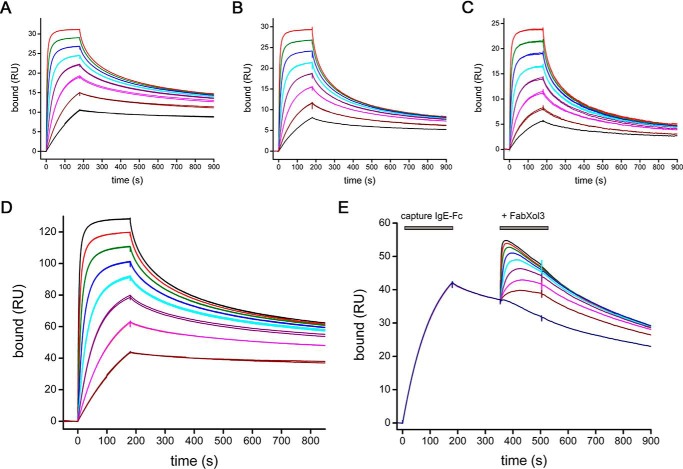
**Interaction studies of FabXol3 with IgE-Fc.** Direct binding was measured for IgE-Fc to immobilized FabXol3 (*A*), FabXol (*B*), and intact omalizumab (*C*). Fabs or intact antibodies were covalently immobilized at low density using an amine coupling kit (GE Healthcare); IgE-Fc was flowed over these surfaces at a variety of concentrations, using a 2-fold dilution series with a highest concentration of 100 nm. *D*, binding of FabXol3 to IgE-Fc captured via a C-terminal His tag; FabXol3 was flowed over IgE-Fc in a 2-fold dilution series with a highest concentration of 100 nm. *E*, binding of the second FabXol3-binding site was characterized using an SPR sandwich binding experiment. IgE-Fc was captured on a FabXol3 surface, and then a second FabXol3 molecule was added to the IgE-Fc/FabXol3 complex in a 2-fold dilution series starting at 1000 nm. For all binding experiments, all concentrations were run in duplicate.

We characterized the IgE-Fc/FabXol3 interaction in two different ways, either by directly immobilizing FabXol3, FabXol (omalizumab Fab), or intact omalizumab on an SPR^5^ sensor surface and binding IgE-Fc, or by binding FabXol3 to His-tagged captured IgE-Fc. The binding characteristics of IgE-Fc to FabXol3, FabXol, and omalizumab were compared (Fig. 7, *A–C*). Not surprisingly, in competition binding experiments, all three molecules competed for the same binding sites and showed broadly similar binding affinities; the FabXol3 construct demonstrates slightly higher affinity compared with FabXol and intact omalizumab (Fig. 7, *A–C*). Consistent with the crystal structure, two FabXol3 molecules bind to IgE-Fc; the binding is clearly biphasic with a high-affinity (∼1 nm) interaction observed at low ligand concentrations and a second (weaker) binding site (∼30 nm) observed at higher concentrations (Fig. 7*D*). It might be speculated that the higher affinity interaction corresponds to the binding of Fab^1^, which would have unimpeded access to a bent IgE-Fc molecule, whereas the lower affinity and slower on-rate corresponds to Fab^2^, but we cannot be definitive about this.

A sandwich-style SPR experiment allowed the two FabXol3-binding sites to be observed and characterized separately. Using this approach, FabXol3 was covalently immobilized on a sensor surface, and IgE-Fc was flowed over this surface. At low concentrations, under these conditions, the high-affinity site dominates the interaction, and the binding curves can be described by monophasic interaction kinetics (*K_D_* ∼1 nm, *k*_on_ ∼1.2 × 10^6^ m^−1^ s^−1^, and *k*_off_ ∼ 8 × 10^−4^ s^−1^). This 1:1 FabXol3/IgE-Fc complex, captured on the SPR biosensor surface, could then be used to measure the binding of the second FabXol3 molecule, the binding of which is significantly weaker (*K_D_* ∼30 nm, *k*_on_ ∼ 2 × 10^5^ m^−1^ s^−1^, and *k*_off_ ∼ 6 × 10^−3^ s^−1^) than the first (Fig. 7*E*). Again, the slower association rate constant measured for the second (weaker) interaction would be consistent with the Fab binding to the less accessible of the two binding sites, *i.e.* the Fab^2^ site in the crystal structure.

**Figure 8.**
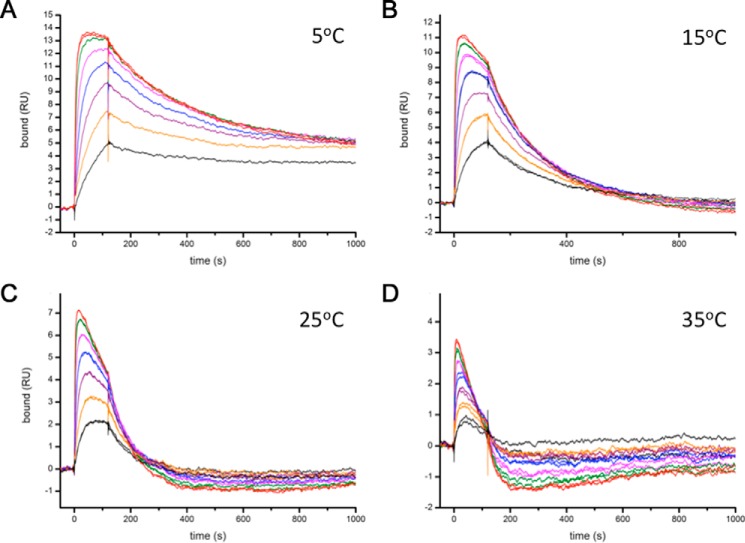
**Temperature dependence of the accelerated dissociation of the IgE-Fc/FabXol3 complex, mediated by the binding of the second FabXol3 molecule.** Binding of the second FabXol3-binding site was characterized using SPR sandwich binding experiments at 5, 15, 25, and 35 °C (*A–D*). IgE-Fc was first captured on a FabXol3 surface and then a second FabXol3 molecule was added to the IgE-Fc/FabXol3 complex, in a 2-fold dilution series starting at 1.6 μm. At low temperature, almost no FabXol3-mediated accelerated dissociation of the IgE-Fc/FabXol3 complex occurs, whereas the effect is markedly increased at higher temperatures.

In characterizing the binding of the two different FabXol3 molecules, we observed that binding of the second FabXol3 molecule destabilized the 1:1 FabXol3/IgE-Fc complex; this destabilization of a pre-formed complex is the same phenomenon of accelerated dissociation that has been seen in IgE in relation to FcϵRI binding (21, 22). We also saw that the FabXol3-mediated accelerated dissociation of the FabXol3/IgE-Fc complex was highly temperature-dependent, with essentially no FabXol3-mediated accelerated dissociation occurring at 5 °C but marked accelerated dissociation occurring at 35 °C (Fig. 8). Because of their physically distal binding sites and the strong temperature dependence of the phenomenon, the ability of the second FabXol3 molecule to mediate accelerated dissociation of the 1:1 FabXol3/IgE-Fc complex must be an allosterically mediated process.

### Competition between the FabXol3- and FcϵRIα-binding sites and the formation of a FabXol3/IgE-Fc/FcϵRIα complex

**Figure 9.**
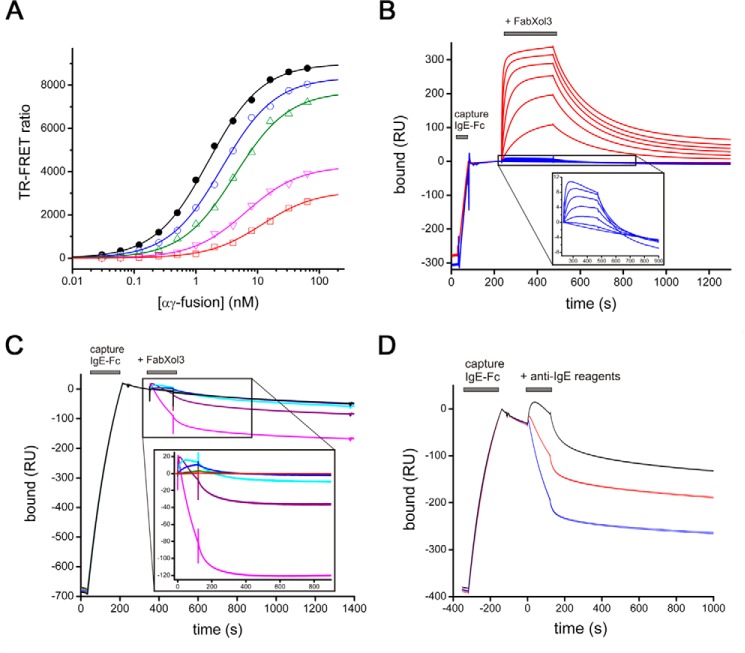
**Analysis of competition binding experiments and accelerated dissociation.** *A*, TR-FRET competition binding experiments between FabXol3 and αγ-fusion protein for IgE-Fc. Binding between terbium-labeled αγ-fusion protein and Alexa Fluor 647-labeled IgE-Fc was measured with increasing concentrations of unlabeled FabXol3 as inhibitor: 0 μm (*black*), 2.5 nm (*blue*), 5 nm (*green*), 10 nm (*magenta*), 20 nm (*red*). As an inhibitor, FabXol3 affects both the apparent *K_D_* and *B*_max_ values of the interaction between IgE-Fc and αγ-fusion protein, indicating some allosteric inhibition properties. *B*, comparison of the ability of FabXol3 to bind to IgE-Fc captured by a C-terminal His tag (*red*) and IgE-Fc captured by binding to sFcϵRIα (*blue*); a 2-fold dilution series was tested for each, starting at 1000 nm. The *inset* shows that FabXol3 can still bind to the IgE-Fc/sFcϵRIα complex, but with a low *B*_max_ value. *C*, accelerated dissociation of the IgE-Fc/sFcϵRIα complex mediated by increasing concentrations of FabXol3. The 1:1 IgE-Fc/sFcϵRIα complex was first established by capturing IgE-Fc on immobilized sFcϵRIα and then binding FabXol3 in a 5-fold dilution series starting at 5000 nm. The *inset* shows a magnification of the accelerated dissociation process. *D*, comparison of the accelerated dissociation of the IgE-Fc/sFcϵRIα complex mediated by intact omalizumab (*black*), FabXol (*red*), or FabXol3 (*blue*), each at a concentration of 5 μm. All binding experiments were performed at 25 °C, except those characterizing the second FabXol3-binding site (*B*), which were performed at 5 °C to minimize allosteric communication between the two sites.

We next investigated the capacity of FabXol3 to affect the interaction between IgE-Fc and FcϵRIα. In solution competition binding experiments, increasing concentrations of FabXol3 inhibited binding of IgE-Fc to FcϵRIα (Fig. 9*A*). Mechanistically, FabXol3 affects both the number of available binding sites (*B*_max_) and the apparent *K_D_* value of the IgE-Fc/FcϵRIα interaction. Reduction in *B*_max_ values is indicative of an allosteric inhibitory process, and a decrease in the apparent affinity of the interaction is most commonly associated with direct competition for a shared binding site (*i.e.* orthosteric inhibition) but can also be seen for some allosteric inhibitors (32). Although we cannot rule out an orthosteric contribution, considering the lack of overlap between the binding sites observed in the crystal structures, it is likely that FabXol3 inhibits IgE-Fc binding to FcϵRI using primarily allosteric mechanisms.

Competition between the omalizumab- and FcϵRIα-binding sites has been described in many publications but was generally interpreted as direct competition between binding sites that were presumed to be identical, or at least overlapping. This interpretation was often used to explain why omalizumab cannot bind to IgE-FcϵRI complexes on cells. We observed, however, that FabXol3 can indeed bind, and with high affinity, to IgE-Fc that is pre-bound to FcϵRIα to form a trimolecular complex (Fig. 9*B*, *inset*). The data indicate that although the binding of IgE-Fc to FcϵRIα did not significantly change the affinity of FabXol3 for IgE-Fc, it did markedly change the number of available binding sites for FabXol3 in the population of FcϵRIα-bound IgE-Fc molecules. We compared the *K_D_* and *B*_max_ binding values for an IgE-Fc molecule captured by an anti-His tag antibody with one captured by sFcϵRIα, and we found that FcϵRIα-bound IgE-Fc had less than 10% of the FabXol3-binding sites compared with the His tag captured IgE-Fc, which, as expected, showed binding levels consistent with 2:1 stoichiometry (Fig. 9*B*). Therefore, it is not that omalizumab does not bind to mast cell-bound IgE because the FcϵRIα and omalizumab-binding sites overlap, or because of steric clashes between two ligands bound to adjacent sites (20). Instead FcϵRIα acts on IgE-Fc allosterically, changing a dynamic equilibrium of different IgE-Fc conformations, resulting in a substantially reduced number of omalizumab-binding sites in a population of FcϵRIα-bound IgE-Fc molecules.

### Mechanism of FabXol3-mediated accelerated dissociation of the IgE-Fc/FcϵRIα complex

Kim *et al.* (21) reported that DARPin E2_79 could accelerate the disassembly of pre-formed complexes of IgE/FcϵRI. Following up on this observation, Eggel *et al.* (22) later showed that omalizumab at high concentrations could also promote dissociation of IgE from FcϵRI. We have found that when FabXol3 binds to the IgE-Fc/sFcϵRIα complex, it accelerates the dissociation of IgE-Fc from sFcϵRIα (Fig. 9*C*) and, furthermore, that FabXol3 does this more efficiently than FabXol, and much more efficiently than intact omalizumab (Fig. 9*D*). One Fab engages the IgE-Fc/sFcϵRIα complex but does not accelerate the dissociation of IgE-Fc from sFcϵRIα. Strikingly, it appears that accelerated dissociation occurs only after occupancy of the second binding site (*i.e.* the low-affinity site). The (FabXol3)_2_/IgE-Fc/sFcϵRIα tetramolecular complex must alter the energy landscape of IgE-Fc in such a way as to markedly reduce the energy barrier for IgE-Fc/sFcϵRIα dissociation, resulting in a rapid dissociation of this otherwise very stable complex.

## Discussion

We report the structure, at 3.7 Å resolution, of the complex between IgE-Fc and a Fab fragment derived from the therapeutic anti-IgE antibody omalizumab; we call this Fab fragment, which contains three point mutations in framework regions distal to the antigen-binding site, FabXol3. The structure reveals two FabXol3 molecules in complex with IgE-Fc, one bound to each of the two Cϵ3 domains, and provides an explanation for the ability of omalizumab to inhibit the binding of IgE to both FcϵRI and CD23. IgE-Fc is also found to adopt a partially bent conformation in the FabXol3 complex, consistent with intramolecular FRET measurements in solution.

IgE-Fc is predominantly bent in solution (4–6, 9, 26–28), and in the crystal structure of free IgE-Fc, the (Cϵ2)_2_ domain pair is folded back against the Cϵ3 and Cϵ4 domains (7, 8). Recently, our understanding of the conformational flexibility of IgE-Fc was profoundly enhanced when we solved the structure of a fully extended conformation, captured in a complex with an anti-IgE-Fc Fab (aϵFab) (16). A molecular dynamics simulation, exploring IgE-Fc unbending from the acutely bent to the extended conformation, revealed energy basins corresponding to partially bent conformations. The FabXol3/IgE-Fc complex reported here, in which the bend between the (Cϵ2)_2_ domain pair and the Fcϵ3–4 domains is ∼90°, corresponds to a distinct energy basin in this simulation (16) (supplemental Fig. S2) and is consistent with our intramolecular FRET measurements (Fig. 5). Intriguingly, the location of the FabXol3-binding site would not preclude further unbending to the fully extended conformation (supplemental Movie S3), and it is therefore possible that IgE-Fc can undergo further substantial changes in conformation even when in complex with omalizumab.

In addition to the bending of the (Cϵ2)_2_ domain pair relative to the Cϵ3 and Cϵ4 domains, the various IgE-Fc, Fcϵ3–4, and receptor complex structures have demonstrated that the Cϵ3 domains can adopt a range of relative orientations, from closed to open (7, 8, 10, 11, 13, 14, 16, 25, 29). Opening and closing of the Cϵ3 domains contributes to the allosteric regulation of receptor binding in IgE-Fc (11, 12); in the CD23 complex they are relatively closed (11, 13, 14), and in the FcϵRI complex they are more open (8, 10). Comparison of the structures of the CD23/Fcϵ3–4 and FabXol3/IgE-Fc complexes shows that the CD23 and omalizumab sites overlap, and competition binding experiments indicate that inhibition of IgE binding to CD23 by omalizumab is straightforwardly orthosteric.

However, inhibition of FcϵRI binding is mechanistically different. In the FabXol3 complex, the Cϵ3 domains adopt a more open conformation than seen in any previous structure, so much so that the two sub-sites of interaction between IgE-Fc and FcϵRI, one involving each Cϵ3 domain, cannot engage simultaneously. In addition to large scale domain motions, local conformational changes induced by FabXol3 binding, such as those in the FcϵRI-binding FG loop, may also contribute to this inhibition. Although there is a possibility of steric clashes if FabXol3 and FcϵRIα bind simultaneously to IgE-Fc, the crystal structure of the FabXol3/IgE-Fc complex demonstrates that omalizumab's mechanism of inhibition is principally allosteric.

SPR experiments to investigate the mechanism of the inhibition of IgE-Fc binding to FcϵRIα by FabXol3 revealed a reduction in the number of available sites for FabXol3 on IgE-Fc (reduced *B*_max_) when in complex with FcϵRIα. The inhibition of IgE binding to FcϵRI by omalizumab has frequently been interpreted in terms of direct competition for overlapping sites, but there have been reports that indicate that omalizumab can bind to receptor-bound IgE (22). Here, we have demonstrated directly the ability of FabXol3 to bind to IgE-Fc when it is already bound to FcϵRIα to form a trimolecular complex. The effect of the pre-binding of IgE-Fc to FcϵRIα is to reduce the number of FabXol3-binding sites on IgE-Fc to less than 10% of those available in free IgE-Fc; this effect can only be due to allosteric modulation.

The nature of the interaction of FabXol3 with the IgE-Fc/FcϵRI complex provides insights into the mechanism of accelerated dissociation of IgE from FcϵRI. This phenomenon was first reported for a DARPin and subsequently for omalizumab (21, 22), the latter at substantially greater concentrations than those used therapeutically (23), and it is now shown here for omalizumab Fab fragments. We further conclude that the dissociation occurs only after binding of the second (lower affinity) FabXol3 molecule. Stated another way, a tetramolecular complex, (FabXol3)_2_/IgE-Fc/FcϵRIα, must be formed for significant accelerated dissociation to occur.

Based on our observations with FabXol3, IgE-Fc, and sFcϵRIα, we envisage the following mechanism occurring for omalizumab, IgE, and FcϵRI. IgE binds to FcϵRI, and under these conditions a small population of the bound IgE molecules adopt a conformation to which omalizumab molecules can bind. When a second omalizumab molecule binds to form the tetrameric complex, the energy landscape of IgE is changed such that the interaction with FcϵRI is destabilized and a rapid dissociation of IgE from FcϵRI occurs.

The inhibitory activities of omalizumab thus take advantage of the intrinsic flexibility of IgE and, for the process of accelerated dissociation, the dynamics of the IgE/FcϵRI complex. IgE has a number of unusual structural characteristics compared with other antibody isotypes, including the presence of the Cϵ2 domains and the uniquely conformationally dynamic molten globule-like character of the Cϵ3 domains (33). Together, these properties create an allosteric communication pathway that prevents simultaneous engagement of CD23 and FcϵRI; this is essential to avoid allergen-independent mast cell activation by cross-linking of FcϵRI-bound IgE by the trimeric CD23 molecule (12). Other functional advantages associated with the dynamics of IgE have been proposed for the membrane-bound IgE B cell receptor (16). We have shown here that omalizumab does not utilize the expected orthosteric mechanism for inhibition of the IgE/FcϵRI interaction, but rather it exploits these unusual dynamic properties of IgE. Furthermore, omalizumab can actively dissociate IgE from FcϵRI, albeit at concentrations higher than used therapeutically, by employing allostery and the intrinsic flexibility of IgE that persists even when in complex with its receptors.

## Experimental procedures

### Cloning, protein expression, and purification

Omalizumab human IgG_1_ Fab, FabXol3, and His-tagged IgE-Fc were cloned, expressed, and purified using methods described in Drinkwater *et al.* (16). IgE-Fc was produced as described previously (34). Omalizumab was purchased from Novartis Europharm Ltd. The 2:1 FabXol3/IgE-Fc complex was purified by size-exclusion chromatography, eluted into 25 mm Tris-HCl, pH 7.5, 20 mm NaCl, and 0.05 (w/v) NaN_3_, and concentrated to 23 mg/ml.

### Surface plasmon resonance

SPR experiments were carried out on a Biacore T200 instrument (GE Healthcare). Specific surfaces were prepared either by covalently coupling proteins using the amine coupling protocol (GE Healthcare), with coupling densities <300 resonance units, or capturing His-tagged proteins using an anti-His sensor surface. For capturing His-tagged ligands, an anti-His tag monoclonal antibody was employed and immobilized according to the manufacturer's instructions (Biacore His Capture Kit, GE Healthcare). In binding experiments, association times of 180–240 s were typically used, and dissociation components were monitored for at least 500 s. Injections were performed at a flow rate of 25 μl min^−1^ in a running buffer of 20 mm HEPES, pH 7.4, 150 mm NaCl, and 0.005% (v/v) surfactant P-20 (GE Healthcare). Most experimental binding measurements were performed at 25 °C; some binding experiments were performed over a range of temperatures (5–35 °C) to control the degree of accelerated dissociation in the system; low temperatures minimize this phenomenon, and higher temperatures increase it. In all cases, standard double referencing data subtraction methods were used (35), and kinetic fits were performed using Origin software (OriginLab).

### TR-FRET

IgE-Fc was labeled with donor fluorophore by reacting 4 mg/ml protein in 100 mm sodium bicarbonate, 50 mm NaCl, pH 9.3, with a 5-fold molar excess of terbium chelate isothiocyanate (Invitrogen). After a 3-h incubation at room temperature with agitation, excess unreacted fluorophore was removed by dialyzing into PBS (20 mm phosphate buffer saline, 150 mm NaCl, pH 7.4). sFcϵRIα-IgG_4_-Fc fusion protein (α-γ) (36) was labeled with acceptor fluorophore by reacting 3 mg/ml protein with a 2.5-fold molar excess of Alexa Fluor 647 succinimidyl ester (Invitrogen) for 1 h at room temperature. Excess fluorophore was removed by dialyzing into PBS.

TR-FRET inhibition assays were performed by competing 1 nm terbium-labeled IgE-Fc and 0–20 nm Alexa Fluor 647-labeled sFcϵRIα-IgG_4_-Fc with a range of concentrations of FabXol3. Assays were conducted in 384-well hi-base white plates (Greiner BioOne) using Lanthascreen buffer (Invitrogen) as a diluent. The plate was left to incubate overnight at room temperature and read by an Artemis plate reader (Berthold Technologies). TR-FRET ratios were then calculated for each well as the emission of acceptor at 665 nm divided by the emission of donor at 620 nm multiplied by 10,000.

### Intramolecular FRET

Measurements of intramolecular FRET were performed essentially as described in Drinkwater *et al.* (16), using an IgE-Fc mutant (E289C) biotinylated at the C terminus using a BirA tag (Avidity). This protein was then fluorescently labeled using a thiol-reactive terbium chelate (Invitrogen) and bound to a monovalent, amine-reactive Alexa Fluor 488 (Invitrogen)-labeled streptavidin (37). The anti-IgE Fabs at various concentrations were added to IgE-Fc in PBS, to give a final concentration of 25 nm IgE-Fc, and incubated for 120 min at 25 °C. FRET was measured on an Analyst HT microplate reader (LJL Biosystems) with an excitation wavelength of 330 nm and emission wavelengths of 485 and 520 nm. Each sample was measured in quadruplicate, and in at least two separate experiments.

### Crystallization

Crystals up to 400 μm in length were grown at 18 °C using the sitting drop vapor diffusion method. The reservoir contained 50 μl of 4% (w/v) PEG 8000 and 0.03 m sodium fluoride, and the drop contained 100 nl of protein solution and 300 nl of reservoir. Despite extensive efforts at optimization, the diffraction quality of the crystals could not be further improved beyond that used for this study. Crystals typically started to grow after a few days and often dissolved in their drops, but they could be stabilized in 4 m trimethylamine *N*-oxide, which was successfully used as a cryoprotectant.

### X-ray data collection and processing

Data were collected at beamlines I02 and I03 at the Diamond Light Source (Harwell, UK). Integration was performed using XDS (38) as implemented in the xia2 package (39). The crystals diffracted anisotropically, and data from multiple crystals were merged. The data were scaled to 3.7 Å resolution with AIMLESS from the CCP4 suite (40, 41) and then truncated to resolution limits of 3.7 Å (a*), 3.9 Å (b*), and 4.2 Å (c*) using the UCLA Diffraction Anisotropy Server (42). Calculation of the Matthews coefficient indicated a solvent content of ∼62%, for a single 2:1 FabXol3/IgE-Fc complex (molecular mass of ∼170 kDa) in the asymmetric unit.

### Structure determination, model building, and refinement

The structure was solved by molecular replacement with PHASER (43) and MOLREP (44) from the CCP4 suite (40) using protein atoms from PDB entry 2WQR (8) and a 1.9 Å resolution omalizumab Fab structure, belonging to the same space group as published crystal structures (19, 24) as search models. Refinement was initially performed with REFMAC (45) and later with PHENIX (46) and alternated with manual model building in Coot (47). The quality of the model was assessed with MolProbity (48) and POLYGON (49). Data processing and refinement statistics are presented in Table 1. A region of the electron density map is shown in supplemental Fig. S4. Interfaces were analyzed with PISA (50), figures were prepared with PyMOL (51), and movies were prepared with Chimera (52), PyMOL (51), and the eMovie plugin (53) for PyMOL.

**Table 1:** **Data processing and refinement statistics**

**Data processing**	
Space group	*I* 2_1_ 2_1_ 2_1_
Unit cell dimensions (Å)	*a* = 76.64, *b* = 231.19, *c* = 247.12
Resolution (Å), overall (outer shell)	115.59–3.70 (4.10–3.70)
Completeness (%)*^a^*	99.9 (99.9)
Multiplicity*^a^*	38.0 (38.4)
Mean ((*I*)/σ(*I*))*^a^*	17.9 (1.9)
*R*_pim_ (%)*^a^*	2.6 (56.3)

**Refinement*^b^***	
*R*_work_/*R*_free_ (%)*^c^*	25.88/30.92
No. of reflections	20 087
Root mean square deviation	
Bond lengths (Å)	0.002
Bond angles (°)	0.451
Coordinate error (Å)	0.60
Average *B*-factor (Å^2^)	171.2
Ramachandran plot	
Favored (%)	95.81
Allowed (%)	100.00

*^a^* Values in parentheses are for the highest resolution shell.

*^b^* Refinement was performed with data truncated to resolution limits of 3.7 Å (a*), 3.9 Å (b*), and 4.2Å (c*).

*^c^ R*_free_ set comprises 5% of reflections.

## Author contributions

T. C., A. J. H., J. M. M., and B. J. S. designed the experiments. A. M. D., E. G. A., A. H. K., J. D., M. O. Y. P., A. J. B., A. J. H., and J. M. M. performed the experiments. A. M. D., E. G. A., A. H. K., J. D., B. P. C. A. N. M., M. O. Y. P., T. C., A. J. B., G. C., M. W., A. J. H., J. M. M., and B. J. S analyzed and/or discussed the data. A. M. D., B. P. C., A. J. H., J. M. M., and B. J. S wrote the manuscript. All authors approved the manuscript.

## Supplementary Material

Supplemental Data

## Footnotes

5 The abbreviations used are:

SPR: surface plasmon resonance
TR-FRET: time resolved-FRET.

## References

[1] Gould H. J., and Sutton B. J. (2008) IgE in allergy and asthma today. Nat. Rev. Immunol. 8, 205–217 18301424 10.1038/nri2273

[2] Sutton B. J., and Davies A. M. (2015) Structure and dynamics of IgE–receptor interactions: FcϵRI and CD23/FcϵRII. Immunol. Rev. 268, 222–235 26497523 10.1111/imr.12340

[3] Holgate S. T. (2014) New strategies with anti-IgE in allergic diseases. World Allergy Organ. J. 7, 17 25097719 10.1186/1939-4551-7-17 PMC4114087

[4] Holowka D., and Baird B. (1983) Structural studies on the membrane-bound immunoglobulin E (IgE)-receptor complex. 2. Mapping of distances between sites on IgE and the membrane surface. Biochemistry 22, 3475–3484 10.1021/bi00283a025 6225455

[5] Zheng Y., Shopes B., Holowka D., and Baird B. (1991) Conformations of IgE bound to its receptor FcϵRI and in solution. Biochemistry 30, 9125–9132 1832555 10.1021/bi00102a002

[6] Beavil A. J., Young R. J., Sutton B. J., and Perkins S. J. (1995) Bent domain structure of recombinant human IgE-Fc in solution by X-ray and neutron scattering in conjunction with an automated curve fitting procedure. Biochemistry 34, 14449–14461 7578050 10.1021/bi00044a023

[7] Wan T., Beavil R. L., Fabiane S. M., Beavil A. J., Sohi M. K., Keown M., Young R. J., Henry A. J., Owens R. J., Gould H. J., and Sutton B. J. (2002) The crystal structure of IgE Fc reveals an asymmetrically bent conformation. Nat. Immunol. 3, 681–686 12068291 10.1038/ni811

[8] Holdom M. D., Davies A. M., Nettleship J. E., Bagby S. C., Dhaliwal B., Girardi E., Hunt J., Gould H. J., Beavil A. J., McDonnell J. M., Owens R. J., and Sutton B. J. (2011) Conformational changes in IgE contribute to its uniquely slow dissociation rate from receptor FcϵRI. Nat. Struct. Mol. Biol. 18, 571–576 21516097 10.1038/nsmb.2044 PMC3357048

[9] Hunt J., Keeble A. H., Dale R. E., Corbett M. K., Beavil R. L., Levitt J., Swann M. J., Suhling K., Ameer-Beg S., Sutton B. J., and Beavil A. J. (2012) A fluorescent biosensor reveals conformational changes in human immunoglobulin E Fc: implications for mechanisms of receptor binding, inhibition, and allergen recognition. J. Biol. Chem. 287, 17459–17470 22442150 10.1074/jbc.M111.331967 PMC3366799

[10] Garman S. C., Wurzburg B. A., Tarchevskaya S. S., Kinet J. P., and Jardetzky T. S. (2000) Structure of the Fc fragment of human IgE bound to its high-affinity receptor FcϵRIα. Nature 406, 259–266 10917520 10.1038/35018500

[11] Dhaliwal B., Yuan D., Pang M. O., Henry A. J., Cain K., Oxbrow A., Fabiane S. M., Beavil A. J., McDonnell J. M., Gould H. J., and Sutton B. J. (2012) Crystal structure of IgE bound to its B-cell receptor CD23 reveals a mechanism of reciprocal allosteric inhibition with high affinity receptor FcϵRI. Proc. Natl. Acad. Sci U.S.A. 109, 12686–12691 22802656 10.1073/pnas.1207278109 PMC3412039

[12] Borthakur S., Hibbert R. G., Pang M. O., Yahya N., Bax H. J., Kao M. W., Cooper A. M., Beavil A. J., Sutton B. J., Gould H. J., and McDonnell J. M. (2012) Mapping of the CD23 binding site on immunoglobulin E (IgE) and allosteric control of the IgE-FcϵRI interaction. J. Biol. Chem. 287, 31457–31461 22815482 10.1074/jbc.C112.397059 PMC3438978

[13] Yuan D., Keeble A. H., Hibbert R. G., Fabiane S., Gould H. J., McDonnell J. M., Beavil A. J., Sutton B. J., and Dhaliwal B. (2013) Ca^2+^-dependent structural changes in the B-cell receptor CD23 increase its affinity for human immunoglobulin E. J. Biol. Chem. 288, 21667–21677 23775083 10.1074/jbc.M113.480657 PMC3724626

[14] Dhaliwal B., Pang M. O., Yuan D., Beavil A. J., and Sutton B. J. (2014) A range of Cϵ3-Cϵ4 interdomain angles in IgE Fc accommodate binding to its receptor CD23. Acta Crystallogr. F Struct. Biol. Commun. 70, 305–309 24598915 10.1107/S2053230X14003355 PMC3944690

[15] Cooper A., and Dryden D. T. (1984) Allostery without conformational change. A plausible model. Eur. Biophys. J. 11, 103–109 6544679 10.1007/BF00276625

[16] Drinkwater N., Cossins B. P., Keeble A. H., Wright M., Cain K., Hailu H., Oxbrow A., Delgado J., Shuttleworth L. K., Kao M. W., McDonnell J. M., Beavil A. J., Henry A. J., and Sutton B. J. (2014) Human immunoglobulin E flexes between acutely bent and extended conformations. Nat. Struct. Mol. Biol. 21, 397–404 24632569 10.1038/nsmb.2795 PMC3977038

[17] Holgate S., Casale T., Wenzel S., Bousquet J., Deniz Y., and Reisner C. (2005) The anti-inflammatory effects of omalizumab confirm the central role of IgE in allergic inflammation. J. Allergy Clin. Immunol. 115, 459–465 15753888 10.1016/j.jaci.2004.11.053

[18] Zheng L., Li B., Qian W., Zhao L., Cao Z., Shi S., Gao J., Zhang D., Hou S., Dai J., Wang H., and Guo Y. (2008) Fine epitope mapping of humanized anti-IgE monoclonal antibody omalizumab. Biochem. Biophys. Res. Commun. 375, 619–622 18725193 10.1016/j.bbrc.2008.08.055

[19] Wright J. D., Chu H. M., Huang C. H., Ma C., Chang T. W., and Lim C. (2015) Structural and physical basis for anti-IgE therapy. Sci. Rep. 5, 11581 26113483 10.1038/srep11581 PMC4481376

[20] Pennington L. F., Tarchevskaya S., Brigger D., Sathiyamoorthy K., Graham M. T., Nadeau K. C., Eggel A., and Jardetzky T. S. (2016) Structural basis of omalizumab therapy and omalizumab-mediated IgE exchange. Nat. Commun. 7, 11610 27194387 10.1038/ncomms11610 PMC4873975

[21] Kim B., Eggel A., Tarchevskaya S. S., Vogel M., Prinz H., and Jardetzky T. S. (2012) Accelerated disassembly of IgE-receptor complexes by a disruptive macromolecular inhibitor. Nature 491, 613–617 23103871 10.1038/nature11546 PMC3504642

[22] Eggel A., Baravalle G., Hobi G., Kim B., Buschor P., Forrer P., Shin J. S., Vogel M., Stadler B. M., Dahinden C. A., and Jardetzky T. S. (2014) Accelerated dissociation of IgE-FcϵRI complexes by disruptive inhibitors actively desensitizes allergic effector cells. J. Allergy Clin. Immunol. 133, 1709–1719 24642143 10.1016/j.jaci.2014.02.005 PMC4083100

[23] Lowe P. J., Tannenbaum S., Gautier A., and Jimenez P. (2009) Relationship between omalizumab pharmacokinetics, IgE pharmacodynamics and symptoms in patients with severe persistent allergic (IgE-mediated) asthma. Br. J. Clin. Pharmacol. 68, 61–76 19660004 10.1111/j.1365-2125.2009.03401.x PMC2732941

[24] Jensen R. K., Plum M., Tjerrild L., Jakob T., Spillner E., and Andersen G. R. (2015) Structure of the omalizumab Fab. Acta Crystallogr. F Struct. Biol. Commun. 71, 419–426 25849503 10.1107/S2053230X15004100 PMC4388177

[25] Cohen E. S., Dobson C. L., Käck H., Wang B., Sims D. A., Lloyd C. O., England E., Rees D. G., Guo H., Karagiannis S. N., O'Brien S., Persdotter S., Ekdahl H., Butler R., Keyes F., et al (2014) A novel IgE-neutralizing antibody for the treatment of severe uncontrolled asthma. MAbs 6, 756–764 24583620 10.4161/mabs.28394 PMC7098617

[26] Zheng Y., Shopes B., Holowka D., and Baird B. (1992) Dynamic conformations compared for IgE and IgG1 in solution and bound to receptors. Biochemistry 31, 7446–7456 1387320 10.1021/bi00148a004

[27] Holowka D., Conrad D. H., and Baird B. (1985) Structural mapping of membrane-bound immunoglobulin-E receptor complexes: use of monoclonal anti-IgE antibodies to probe the conformation of receptor-bound IgE. Biochemistry 24, 6260–6267 2935182 10.1021/bi00343a033

[28] Davis K. G., Glennie M., Harding S. E., and Burton D. R. (1990) A model for the solution conformation of rat IgE. Biochem. Soc. Trans. 18, 935–936 2083747 10.1042/bst0180935

[29] Wurzburg B. A., and Jardetzky T. S. (2009) Conformational flexibility in immunoglobulin E-Fc_3–4_ revealed in multiple crystal forms. J. Mol. Biol. 393, 176–190 19682998 10.1016/j.jmb.2009.08.012 PMC2827403

[30] Shiung Y. Y., Chiang C. Y., Chen J. B., Wu P. C., Hung A. F., Lu D. C., Pan R. L., and Chang T. W. (2012) An anti-IgE monoclonal antibody that binds to IgE on CD23 but not on high-affinity IgE. Fc receptors. Immunobiology 217, 676–683 22226669 10.1016/j.imbio.2011.11.006

[31] Borthakur S., Andrejeva G., and McDonnell J. M. (2011) Basis of the intrinsic flexibility of the Cϵ3 domain of IgE. Biochemistry 50, 4608–4614 21520934 10.1021/bi200019y

[32] Fersht A (1999) Structure and Mechanism in Protein Science, pp. 103–131, W. H. Freeman & Co., New York

[33] Price N. E., Price N. C., Kelly S. M., and McDonnell J. M. (2005) The key role of protein flexibility in modulating IgE interactions. J. Biol. Chem. 280, 2324–2330 15520005 10.1074/jbc.M409458200

[34] Young R. J., Owens R. J., Mackay G. A., Chan C. M., Shi J., Hide M., Francis D. M., Henry A. J., Sutton B. J., and Gould H. J. (1995) Secretion of recombinant human IgE-Fc by mammalian cells and biological activity of glycosylation site mutants. Protein Eng. 8, 193–199 7543206 10.1093/protein/8.2.193

[35] Myszka D. G. (1999) Improving biosensor analysis. J. Mol. Recognit. 12, 279–284 10556875 10.1002/(SICI)1099-1352(199909/10)12:5<279::AID-JMR473>3.0.CO;2-3

[36] Shi J., Ghirlando R., Beavil R. L., Beavil A. J., Keown M. B., Young R. J., Owens R. J., Sutton B. J., and Gould H. J. (1997) Interaction of the low-affinity receptor CD23/FcϵRII lectin domain with the Fcϵ3–4 fragment of human immunoglobulin E. Biochemistry 36, 2112–2122 9047310 10.1021/bi961231e

[37] Howarth M., and Ting A. Y. (2008) Imaging proteins in live mammalian cells with biotin ligase and monovalent streptavidin. Nat. Protoc. 3, 534–545 18323822 10.1038/nprot.2008.20 PMC2671200

[38] Kabsch W. (2010) XDS. Acta Crystallogr. D Biol. Crystallogr. 66, 125–132 20124692 10.1107/S0907444909047337 PMC2815665

[39] Winter G. (2010) xia2: an expert system for macromolecular crystallography data reduction. J. Appl. Crystallogr. 43, 186–190

[40] Winn M. D., Ballard C. C., Cowtan K. D., Dodson E. J., Emsley P., Evans P. R., Keegan R. M., Krissinel E. B., Leslie A. G., McCoy A., McNicholas S. J., Murshudov G. N., Pannu N. S., Potterton E. A., Powell H. R., et al (2011) Overview of the CCP4 suite and current developments. Acta Crystallogr. D Biol. Crystallogr. 67, 235–242 21460441 10.1107/S0907444910045749 PMC3069738

[41] Evans P. R., and Murshudov G. N. (2013) How good are my data and what is the resolution? Acta Crystallogr. D Biol. Crystallogr. 69, 1204–1214 23793146 10.1107/S0907444913000061 PMC3689523

[42] Strong M., Sawaya M. R., Wang S., Phillips M., Cascio D., and Eisenberg D. (2006) Toward the structural genomics of complexes: crystal structure of a PE/PPE protein complex from *Mycobacterium tuberculosis*. Proc. Natl. Acad. Sci. U.S.A. 103, 8060–8065 16690741 10.1073/pnas.0602606103 PMC1472429

[43] McCoy A. J., Grosse-Kunstleve R. W., Adams P. D., Winn M. D., Storoni L. C., and Read R. J. (2007) Phaser crystallographic software. J. Appl. Crystallogr. 40, 658–674 19461840 10.1107/S0021889807021206 PMC2483472

[44] Vagin A., and Teplyakov A. (2010) Molecular replacement with MOLREP. Acta Crystallogr. D Biol. Crystallogr. 66, 22–25 20057045 10.1107/S0907444909042589

[45] Murshudov G. N., Skubák P., Lebedev A. A., Pannu N. S., Steiner R. A., Nicholls R. A., Winn M. D., Long F., and Vagin A. A. (2011) REFMAC5 for the refinement of macromolecular crystal structures. Acta Crystallogr. D Biol. Crystallogr. 67, 355–367 21460454 10.1107/S0907444911001314 PMC3069751

[46] Afonine P. V., Grosse-Kunstleve R. W., Echols N., Headd J. J., Moriarty N. W., Mustyakimov M., Terwilliger T. C., Urzhumtsev A., Zwart P. H., and Adams P. D. (2012) Towards automated crystallographic structure refinement with phenix.refine. Acta Crystallogr. D Biol. Crystallogr. 68, 352–367 22505256 10.1107/S0907444912001308 PMC3322595

[47] Emsley P., Lohkamp B., Scott W. G., and Cowtan K. (2010) Features and development of Coot. Acta Crystallogr. D Biol. Crystallogr. 66, 486–501 20383002 10.1107/S0907444910007493 PMC2852313

[48] Chen V. B., Arendall W. B. 3rd., Headd J. J., Keedy D. A., Immormino R. M., Kapral G. J., Murray L. W., Richardson J. S., and Richardson D. C. (2010) MolProbity: all-atom structure validation for macromolecular crystallography. Acta Crystallogr. D Biol. Crystallogr. 66, 12–21 20057044 10.1107/S0907444909042073 PMC2803126

[49] Urzhumtseva L., Afonine P. V., Adams P. D., and Urzhumtsev A. (2009) Crystallographic model quality at a glance. Acta Crystallogr. D Biol. Crystallogr. 65, 297–300 19237753 10.1107/S0907444908044296 PMC2651759

[50] Krissinel E., and Henrick K. (2007) Inference of macromolecular assemblies from crystalline state. J. Mol. Biol. 372, 774–797 17681537 10.1016/j.jmb.2007.05.022

[51] DeLano W. L. (2010) The PyMOL Molecular Graphics System, Version 1.1r1, Schrödinger, LLC, New York

[52] Pettersen E. F., Goddard T. D., Huang C. C., Couch G. S., Greenblatt D. M., Meng E. C., and Ferrin T. E. (2004) UCSF Chimera–a visualization system for exploratory research and analysis. J. Comput. Chem. 25, 1605–1612 15264254 10.1002/jcc.20084

[53] Hodis E., Schreiber G., Rother K., and Sussman J. L. (2007) eMovie: a storyboard-based tool for making molecular movies. Trends Biochem. Sci. 32, 199–204 17448663 10.1016/j.tibs.2007.03.008

