# Supplementary material for: Allosteric mechanism of action of the therapeutic anti-IgE antibody omalizumab
Source: J Biol Chem. 2017 Apr 24;292(24):9975–87. doi: 10.1074/jbc.M117.776476 (PMC5473249; doi:10.1074/jbc.M117.776476)
Supplement: Supplemental Data [file supp_292_24_9975__index.html]

Allosteric mechanism of action of the therapeutic anti-IgE antibody omalizumab — Allosteric mechanism of action of the therapeutic anti-IgE antibody omalizumab — Allosteric mechanism of action of omalizumab — Supplemental Data 

# Allosteric mechanism of action of the therapeutic anti-IgE antibody omalizumab

## Supplemental Data

- Movie S1 (.avi, 6.0 MB) - Supplemental Movie S1. Overall structure of the FabXol3/IgE-Fc complex.
- Movie S2 (.avi, 8.0 MB) - Supplemental Movie S2. Conformational changes in IgE-Fc.
- Movie S3 (.avi, 5.6 MB) - Supplemental Movie S3. IgE-Fc is potentially conformationally flexible when in complex with FabXol3/omalizumab.
- Supplemental Data (.doc, 2.6 MB) - Supplemental Data
