# Supplementary material for: Allosteric mechanism of action of the therapeutic anti-IgE antibody omalizumab
Source: J Biol Chem. 2017 Apr 24;292(24):9975–87. doi: 10.1074/jbc.M117.776476 (PMC5473249; doi:10.1074/jbc.M117.776476)
Supplement: Supplemental Data [file 10.1074_M117.776476_jbc.M117.776476-4.doc]

**SUPPLEMENTAL DATA**

Allosteric mechanism of action of the therapeutic anti-IgE antibody omalizumab

**Anna M. Davies^1,2^, Elizabeth G. Allan^1,2^, Anthony H. Keeble^1,2^, Jean Delgado^3^, Benjamin P. Cossins^3^, Alkistis N. Mitropoulou^1,2^, Marie O.Y. Pang^1,2^, Tom Ceska^3^, Andrew J. Beavil^1,2^, Graham Craggs^3^, Marta Westwood^3^, Alistair J. Henry^3^, James M. McDonnell^1,2^, and Brian J. Sutton^1,2^**

**CONTENTS**

**1. Supplemental text**

Details of the interaction between the FabXol3 light chain and the Cε2 domain.

Analysis of Cε3 domain orientation in the FabXol3/IgE-Fc complex.

**2. Figure S1.** FabXol3 contains three point mutations.

**2. Figure S2.** Conformational flexibility in IgE-Fc.

**3. Figure S3.** The Cε3 domains adopt a markedly open conformation in the FabXol3/IgE-Fc complex.

**4. Figure S4.** Representative electron density map.

**5. Movie captions**

**Movie S1.** Overall structure of the FabXol3/IgE-Fc complex

**Movie S2.** Conformational changes in IgE-Fc

**Movie S3.** IgE-Fc is potentially conformationally flexible when in complex with FabXol3/omalizumab

**6. References**

**SUPPLEMENTAL TEXT**

***Details of the interaction between the FabXol3 light chain and the Cε2 domain***

In the FabXol3/IgE-Fc complex, one Cε2 domain forms a minor interaction of approximately 260Å^2^ (compared with an average interaction area of ~715Å^2^ between FabXol3 and the Cε3 domain) with two of the mutated residues (Ser81Arg and Gln83Arg). There is no contact between Pro158 and IgE-Fc.

The Arg81 side chain, from the Fab^2^ light chain (one of the mutated residues in FabXol3), packs against Val277 and Asp278 from the Cε2 domain from chain B. Ser80 (FabXol3) packs against Asp278, Leu279 and Thr281 (Cε2 domain), while Ser64 (FabXol3) packs against Asp276 and Asp278. Ser64 and Ser80 are identical in omalizumab and FabXol3.

In the FabXol3/IgE-Fc complex, Arg83 (one of the mutated residues in aεFabXol3) does not apparently contact the Cε2 domain, due to disorder in the Asp278 (Cε2 domain) side chain. However, if the Asp278 side chain were ordered, a hydrogen bond or salt bridge could potentially form between Arg83 and Asp278.

***Analysis of Cε3 domain orientation in the FabXol3/IgE-Fc complex***

In one method to analyse the position of the Cε3 domains with respect to the Cε4 domains, the interatomic distance between the Asn394 Cα atom from the Cε3 domain of one chain, and the Lys497 Cα atom from the Cε4 domain of the other chain has been used to describe the “openness” of the Cε3 domains (1). The interatomic distance between the Val336 Cα has been used to describe the “swing”, or how close the Cε3 domains are to one another (1).

For FcεRI-bound IgE-Fc, and FcεRI-bound Fcε3-4, in which the Cε3 domains adopt an open conformation, “openness” values range from 23.5-28.4Å, while the “swing” values are an average of 23.3Å (2,3). Corresponding values for the FabXol3/IgE-Fc complex are an average of 30.2 Å for the “openness” and 29.3Å for the “swing”. In the FabXol3 complex, the Cε3 domains adopt the most open conformation (furthest apart from one another), described thus far (Fig. S3).

**
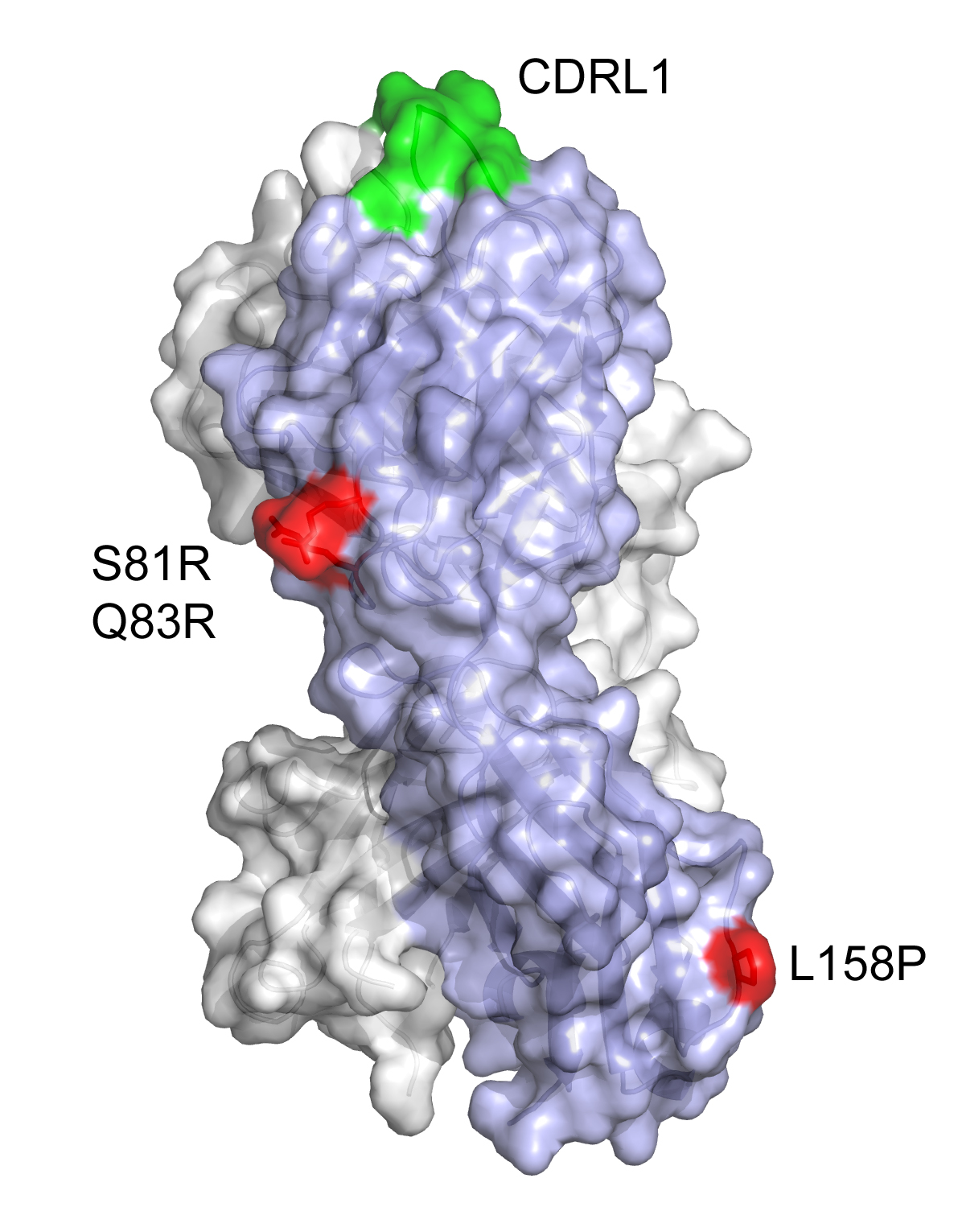
**

**Figure S1**. **FabXol3 contains three point mutations.** FabXol3 is derived from omalizumab, and contains three point mutations distal to the antigen binding CDRs, two in the V_L_ domain framework region (Ser81Arg, Gln83Arg) and one in the Cκ domain (Leu158Pro). The heavy and light chains are coloured white and blue, respectively. The mutated residues are coloured in red, and CRDL1 in green, to indicate the orientation of the Fab.

**
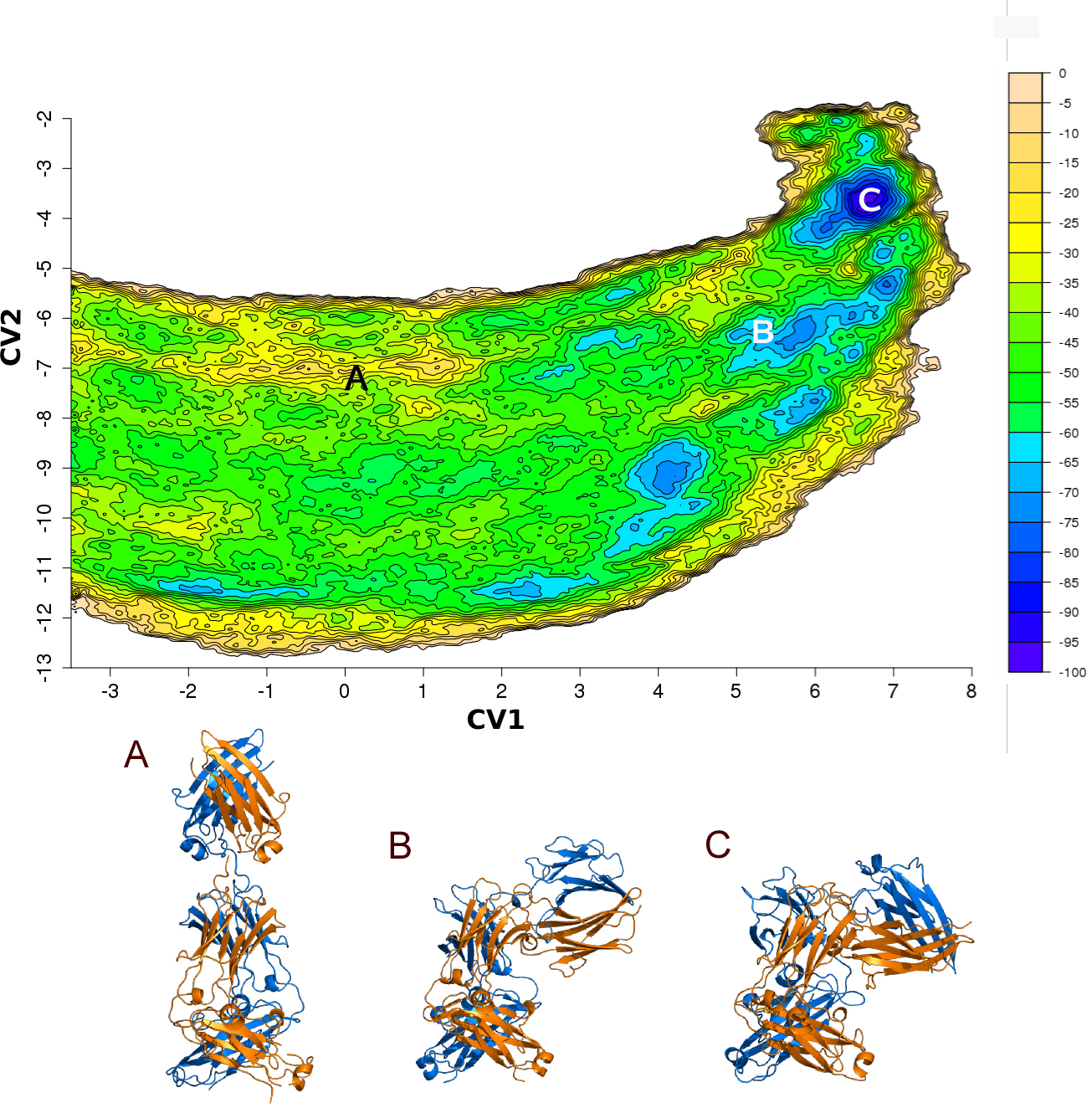
**

**Figure S2: Conformational flexibility in IgE-Fc.** Flexibility of IgE-Fc, and unbending from the bent to a fully extended conformation, was previously explored by molecular dynamics (4). IgE-Fc unbending is represented as a free energy surface, as previously described (4). (A) Extended conformation of IgE-Fc captured in the crystal structure of the aεFab/IgE-Fc complex (4). (B) Partially bent IgE-Fc conformation observed in the crystal structure of the FabXol3/IgE-Fc complex. (C) Bent conformation of free IgE-Fc (2,5). The bent conformation of IgE-Fc occupies the lowest energy basin, while the partially bent conformation observed in the FabXol3/IgE-Fc complex occupies a clearly distinct energy basin (B). Adapted by permission from Macmillan Publishers Ltd: [Nature Structural and Molecular Biology], (Drinkwater, N. et al., (2014) Human immunoglobulin E flexes between acutely bent and extended conformations, Nature Structural and Molecular Biology 21,397-404), copyright (2014).

**
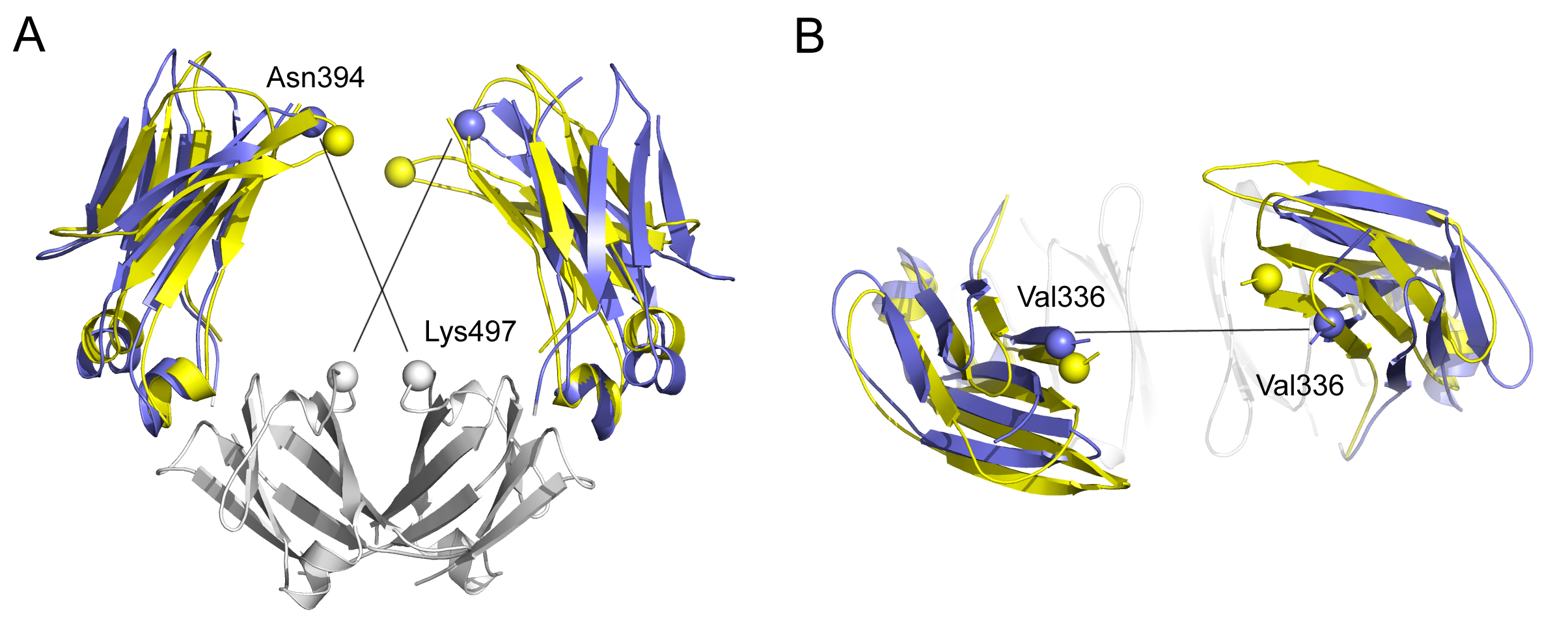
**

**Figure S3: The Cε3 domains adopt a markedly open conformation in the FabXol3/IgE-Fc complex.** (A) The Fcε3-4 region from the FabXol3 and sFcεRIα (2) complexes with IgE-Fc were superposed on the Cε4 domains. The Cε4 domains for the FabXol3 complex are coloured gray, and the Cε3 domains from the FabXol3 and sFcεRIα complexes are coloured blue and yellow, respectively. The Cε3 domains adopt a more “open” conformation in the FabXol3 complex, compared with the sFcεRIα complex. (B) In the FabXol3 complex (blue), the Cε3 domains are further apart from one another compared with the sFcεRIα complex (yellow).

**
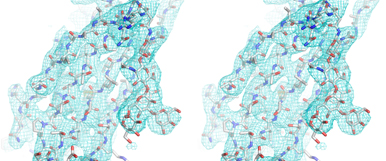
**

**Figure S4. Representative electron density map.** A stereoview of the 2F_o_-F_c_ electron density map, contoured at 1.1σ, is shown for a portion of the chain A Cε3 domain, and covalently N-linked oligosaccharide moiety at Asn394.

**MOVIE CAPTIONS**

**Movie S1. Overall structure of the FabXol3/IgE-Fc complex.**

The movie depicts a surface representation of the complex. IgE-Fc chains A and B are coloured in yellow and pink respectively, while the two FabXol3 molecules are coloured in blue and green. The structure is first rotated 90° towards the viewer to illustrate the complex from the perspective of the (Cε2)_2_ domain pair at the “top” of the structure. The structure is rotated 90° away from the viewer, back to the starting position, and then rotated 360° about an axis parallel to the pseudo two-fold axis of the Cε3 and Cε4 domains.

**Movie S2. Conformational changes in IgE-Fc.** The movie begins by showing a surface representation of free IgE-Fc. The structure is morphed from the bent conformation of free IgE-Fc to the partially bent conformation observed in the FabXol3/IgE-Fc complex, and then back to the bent conformation. The structure is rotated 360° about an axis parallel to the pseudo two-fold axis of the Fcε3-4 region, pausing at 90° intervals to show the morph. The view of the structure is then zoomed out, and the structure rotated to orient the Cε2 domains towards the viewer. The two Fab molecules are shown binding to IgE-Fc after morphing from the bent conformation to the partially bent conformation in the FabXol3 complex. IgE-Fc chains A and B are coloured in yellow and pink respectively, while the two Fab molecules are coloured in green and blue. Binding of both Fab molecules to IgE-Fc in the final morph is not intended to represent the mechanism of IgE-Fc engagement by FabXol3.

**Movie S3. IgE-Fc is potentially conformationally flexible when in complex with FabXol3/omalizumab.** The movie begins by showing a surface representation of the FabXol3/IgE-Fc complex. The conformation of IgE-Fc is then morphed from the partially bent conformation observed in the FabXol3 complex to the fully extended conformation observed in the aεFab complex. The structure is rotated 360° about an axis parallel to the pseudo two-fold axis of the IgE-Cε3 and Cε4 domains, pausing at 90° intervals to show the morph, revealing that IgE-Fc is potentially conformationally dynamic when in complex with FabXol3/omalizumab. IgE-Fc chains A and B are coloured in yellow and pink respectively, while the two Fabs are coloured in green and blue.

**REFERENCES**

1. Wurzburg, B. A., and Jardetsky, T. S. (2009) Conformational flexibility in immunoglobulin E- Fc_3–4_ revealed in multiple crystal forms. *J. Mol. Biol*. **393,** 176-190

2. Holdom, M. D., Davies, A. M., Nettleship, J. E., Bagby, S. C., Dhaliwal, B., Girardi, E., Hunt, J., Gould, H. J., Beavil, A. J., McDonnell, J. M., Owens, R. J., and Sutton, B. J. (2011) Conformational changes in IgE contribute to its uniquely slow dissociation rate from receptor FcɛRI. *Nat. Struct. Mol. Biol*. **18,** 571-576

3. Garman, S. C., Wurzburg, B. A., Tarchevskaya, S. S., Kinet, J. P., and Jardetzky, T. S. (2000) Structure of the Fc fragment of human IgE bound to its high-affinity receptor FcεRIα. *Nature* **406,** 259-266

4. Drinkwater, N., Cossins, B. P., Keeble, A. H., Wright, M., Cain, K., Hailu, H., Oxbrow, A., Delgado, J., Shuttleworth, L. K., Kao, M. W., McDonnell, J. M., Beavil, A. J., Henry, A. J., and Sutton, B. J. (2014) Human immunoglobulin E flexes between acutely bent and extended conformations. *Nat. Struct. Mol. Biol.* **21,** 397-404

5. Wan, T., Beavil, R. L., Fabiane, S. M., Beavil, A. J., Sohi, M. K., Keown, M., Young, R. J., Henry, A. J., Owens, R. J., Gould, H. J., and Sutton, B. J. (2002) The crystal structure of IgE Fc reveals an asymmetrically bent conformation. *Nat. Immunol*. **3,** 681-686
